# Vasorelaxant Effects of Wild Jujube Leaf Aqueous Extract: Endothelial NO/cGMP/SERCA Signaling, Antioxidant Activity, and AI-Based Molecular Docking

**DOI:** 10.3390/molecules31183252

**Published:** 2026-09-14

**Authors:** Chaimae Alla, Zachée Louis Evariste Akissi, Moulay Hfid Youssoufi, Afaf Mehiou, Amanat Ali, Ikram Dib, Nabia El-aouni, Hassane Mekhfi, Abdelkhaleq Legssyer, Sergey Shityakov, Abderrahim Ziyyat, Sevser Sahpaz

**Affiliations:** 1 Laboratory of Pharmacology, Genomics, Environment and Health, Department of Biology, Faculty of Sciences, Mohammed First University, Oujda 62000, Morocco; chaimae.alla@ump.ac.ma (C.A.); afaf.mehiou@ump.ac.ma (A.M.); amanata@gmail.com (A.A.); i.dib@ump.ac.ma (I.D.); nabia.elaouni789@gmail.com (N.E.-a.); h.mekhfi@ump.ac.ma (H.M.); a.legssyer@ump.ac.ma (A.L.); ar.ziyyat@ump.ac.ma (A.Z.); 2 BioEcoAgro Joint Research Unit (UMRT), University of Lille, INRAE, Liège, UPJV, JUNIA, Artois, Littoral Côte d’Opale, 59000 Lille, France; zachee.akissi@univ-lille.fr; 3 Laboratory of Applied Chemistry and Environment, Faculty of Sciences, Mohammed First University, Oujda 62000, Morocco; hafid.youssoufi@gmail.com; 4 Laboratory of Chemoinformatics, Infochemistry Scientific Center, ITMO University, 191002 St. Petersburg, Russia; shityakoff@hotmail.com; 5 Department of Biology and General Genetics, Sechenov First Moscow State Medical University (Sechenov University), 119992 Moscow, Russia

**Keywords:** *Ziziphus lotus*, hypertension, vasorelaxation, endothelial signaling pathway, molecular docking analysis

## Abstract

*Ziziphus lotus* (L.) Lam (wild jujube) leaves are traditionally used in Moroccan medicine to treat hypertension. The present study evaluated the aqueous extract of *Z. lotus* leaves (ZLAqExt) for its vasorelaxant effects, mechanisms of action, acute toxicity, antioxidant potential, and phytochemical profile. In phenylephrine-precontracted rat aortic rings, the ZLAqExt induced concentration-dependent relaxation (Emax = 79.25 ± 2.77% at 10^−1^ mg/mL). The mechanism of action was investigated using specific antagonists and blockers. Complete inhibition by endothelium denudation, and preincubation with L-NAME, hydroxocobalamin, ODQ, and thapsigargin confirmed endothelium-dependent nitric oxide (NO)/cyclic GMP (cGMP)/sarco/endoplasmic reticulum Ca^2+^-ATPase (SERCA) pathway involvement. A partial inhibition by BaCl_2_, indomethacin, calmidazolium, and KCl indicated cyclooxygenase (COX), endothelium-derived hyperpolarizing factor (EDHF), and inward-rectifier potassium (K_ir_) channel contributions. Acute oral toxicity did not show any mortality at 2 g/kg BW in mice. The extract displayed potent antioxidant activity (DPPH IC_50_: 18.12 ± 1.49 µg/mL; β-carotene bleaching IC_50_: 8.3 ± 0.014 µg/mL). UHPLC-ESI-MS identified rutin as the major compound, followed by 3′,5′-di-C-β-glucopyranosyl phloretin with high docking affinity for guanylate cyclase and SERCA pump. The presence of flavonoids in the ZLAqExt might have mediated its vasorelaxant effects, supporting its traditional use as an antihypertensive. Our results highlight that *Z. lotus* is a potential natural therapeutic agent against vascular dysfunction-related cardiovascular disorders.

## 1. Introduction

Cardiovascular diseases rank as the top global killer, surpassing other pathological states [1,2]. Hypertension constitutes a principal determinant in this mortality burden [3]. Although various categories of antihypertensive pharmaceutical agents are accessible [4], their long-term administration frequently elicits adverse reactions that impact 20% to 97% of treated individuals [5]. This realization accentuates the need for antihypertensive strategies characterized by improved safety and durability profiles. Based on an international scale, the botanical remedies are utilized for the management of numerous pathologies due to their inherent bioactive characteristics. It is estimated that almost 80% of the inhabitants within developing countries rely on traditional therapeutic modalities as foundational healthcare provisions [6,7]. Phytochemical-based derivatives comprise at least 25% of the pharmacopoeia in industrialized countries and facilitate the advancement of innovative therapeutic strategies [8]. Appropriate modulation of vascular smooth muscle preserves the blood pressure homeostasis; however, the irregularities affecting this modulatory capacity may precipitate hypertensive conditions [9]. Natural derivative agents present encouraging prospects for vascular regulation directed at hypertension prevention and management [10].

*Z. lotus* (L.) Lam. (Rhamnaceae) is a shrub indigenous to the Mediterranean basin. It is widely distributed across North Africa and the Middle East territories and is valued for its therapeutic properties [11]. In the traditional medicine of Morocco, Algeria, and Mauritania, it serves as a common remedy for hypertension [12,13,14]. Pharmacological studies confirm that the extracts from its fruit, leaves, roots, and other parts exhibit antioxidant, anti-inflammatory, analgesic, antimicrobial, antidiabetic, antihyperlipidemic, antitumoral, antiulcerogenic, antispasmodic, and antilithiatic properties [15]. Based on the current state of knowledge, however, the effects of its aqueous leaf extract on vascular tone regulation have not yet been investigated. The present research, therefore, focused on assessing the vasorelaxant effects and underlying mechanisms of the aqueous leaf extract of *Z. lotus* (L.) Lam (ZLAqExt), along with its antioxidant activities, acute toxicity, and phytochemical profile. Molecular docking simulations further explored the molecular interactions between the ZLAqExt’s key compounds and their vasorelaxant targets.

## 2. Results

### 2.1. Vasorelaxation Response in Normotensive Rat Aorta

#### 2.1.1. Endothelial Modulation of ZLAqExt-Induced Vasorelaxation in PHE-Constricted Aortic Segments

**Figure 1:**
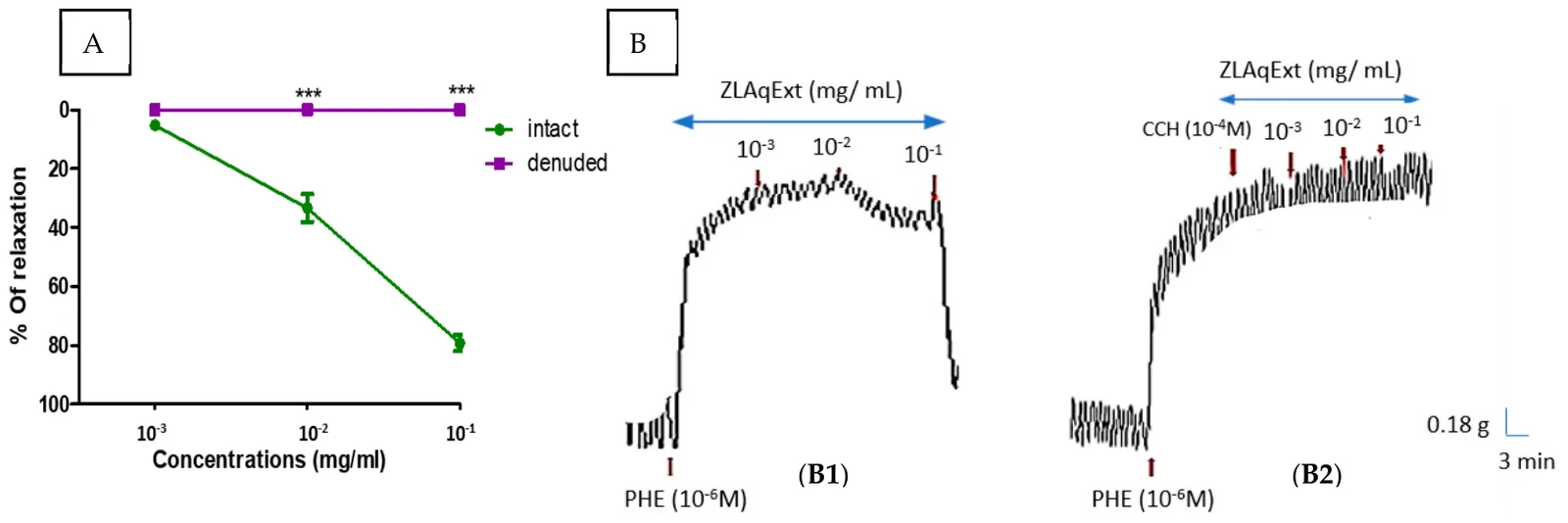
Panel (**A**): Concentration-response curves illustrating the vasorelaxant effect of ZLAqExt on endothelium-intact and -denuded aortic rings pre-contracted with PHE (10^−6^ M). Results are reported as mean ± SEM (*n* = 6), analysed via two-way ANOVA/Bonferroni, *** *p* < 0.001 vs. intact rings. Panel (**B**): Original recordings showing the vascular effect of ZLAqExt on intact (**B1**) and denuded (**B2**) aortic rings. Red arrow indicates the moment of injection.

The aqueous extract of *Z. lotus* (L.) Lam. (ZLAqExt) elicited notable vasorelaxation in PHE-precontracted aortic rings from rats (*n* = 6) with an intact endothelium. The aortic rings exhibited a concentration-dependent relaxation in response to ZLAqExt at concentrations of 10^−3^, 10^−2^, and 10^−1^ mg/mL. The extract yielded an effective concentration (EC_50_) of 0.036 ± 0.012 mg/mL against PHE-induced contraction, with a corresponding Emax of 79.25 ± 2.77% at 10^−1^ mg/mL (Figure 1A,B). However, the relaxing effect of CCH and ZLAqExt on isolated aortic rings was completely abolished after mechanical disruption of the endothelium.

#### 2.1.2. Involvement of the Ca^2+^-Calmodulin/NO/sGC Pathway and Muscarinic Receptors in ZLAqExt-Induced Vasorelaxation

**Figure 2:**
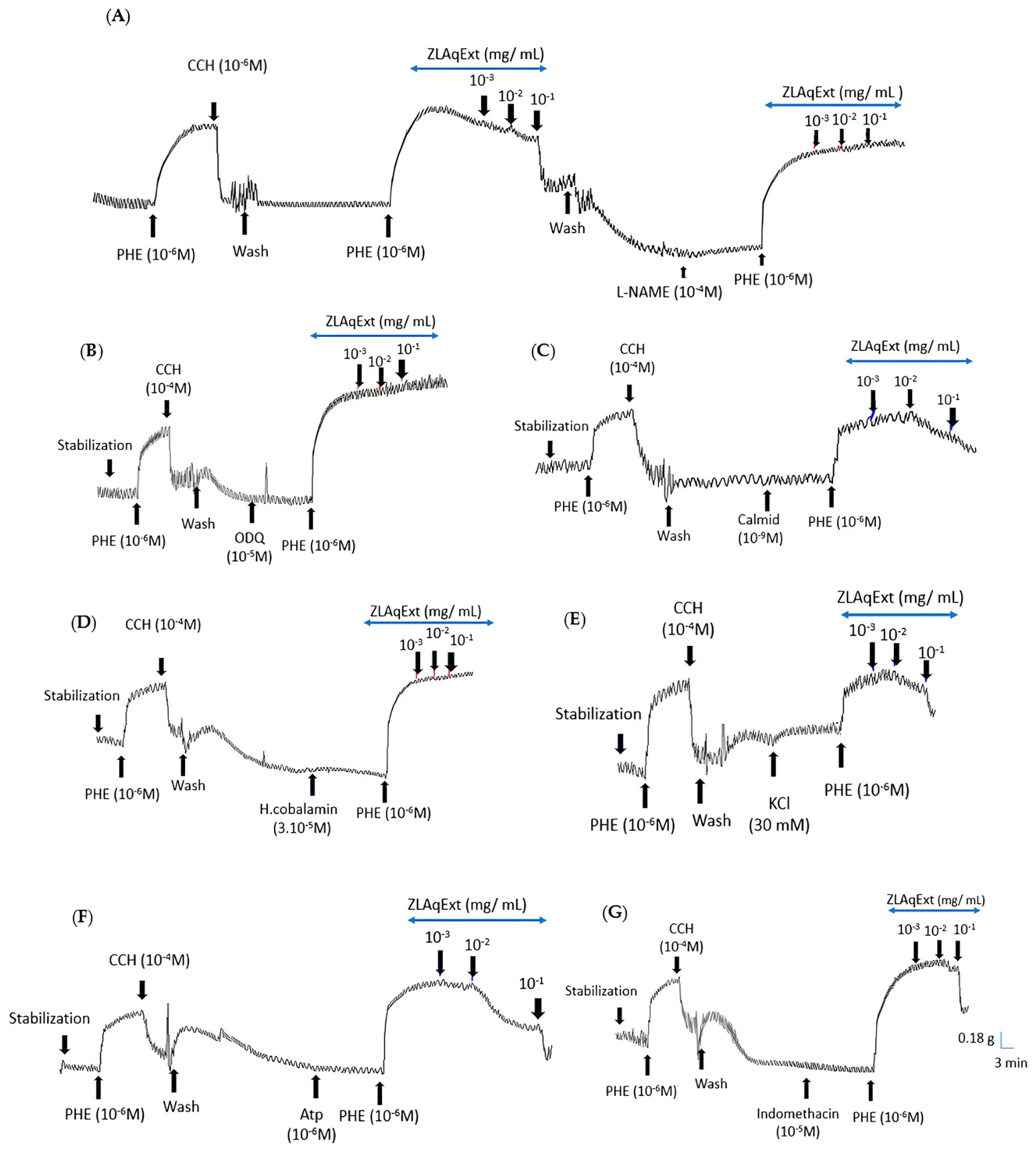
Original recordings demonstrate the vascular effect of ZLAqExt in intact aortic rings treated with (**A**) L-NAME, (**B**) ODQ, (**C**) calmidazolium, (**D**) hydroxocobalamin, (**E**) KCl, (**F**) atropine, and (**G**) indomethacin. The black arrow indicates the moment of injection.

**Figure 3:**
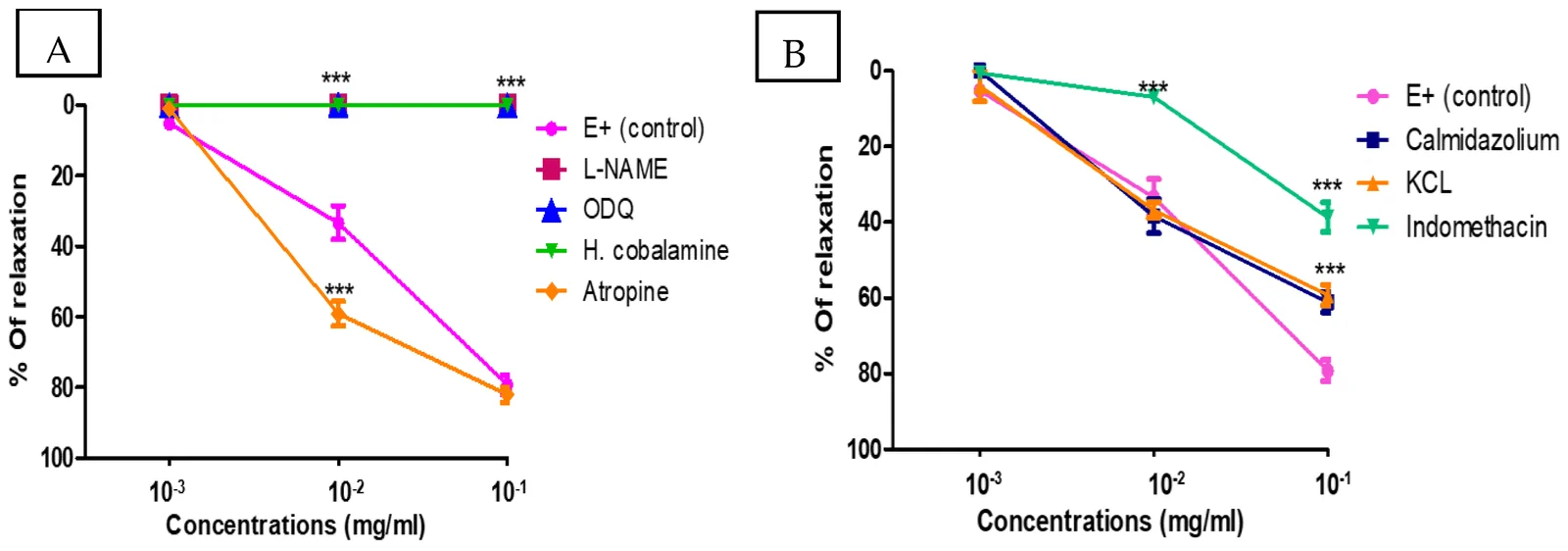
Vasorelaxant-response curves of ZLAqExt in the presence of various pharmacological inhibitors: (Panel (**A**)) L-NAME, ODQ, hydroxocobalamin, and atropine; (Panel (**B**)) calmidazolium, KCl, and indomethacin. Results are presented as mean ± SEM (*n* = 6 aortic rings/12 rats). Statistical significance was assessed using two-way ANOVA with Bonferroni post hoc test. *** *p* < 0.001 vs. control (endothelium-intact, E+).

The vasorelaxant effect of ZLAqExt was completely abolished in endothelium-intact aortic rings pretreated with the eNOS inhibitor L-NAME, the sGC inhibitor ODQ, or the NO scavenger hydroxocobalamin (Figure 2 and Figure 3A). Conversely, when tissues were pretreated with the selective muscarinic receptor antagonist atropine, a similar vasorelaxant effect was maintained, with an Emax of 82.08 ± 2.19% (*p* > 0.05, *n* = 6). However, the inhibition was less pronounced with the Ca^2+^-calmodulin antagonist calmidazolium (Emax = 61.22 ± 2.64%; *p* < 0.001, *n* = 6) (Figure 2 and Figure 3B).

#### 2.1.3. Involvement of the COX and EDHF Pathways in ZLAqExt-Induced Vasorelaxation

Pre-incubation with indomethacin (a non-selective COX inhibitor) significantly blunted the ZLAqExt-induced vasorelaxation, limiting the maximal response to 38.75 ± 3.84% (*p* < 0.001, *n* = 6). Furthermore, the relaxation was partially impaired in the presence of KCl, suggesting a role for endothelium-derived hyperpolarizing factors (Emax = 59.32 ± 2.69%; *p* < 0.001, *n* = 6) (Figure 2 and Figure 3B).

#### 2.1.4. Involvement of Potassium Channels (K_IR_, K_V_, K_Ca_, and K_ATP_) in ZLAqExt-Induced Vasorelaxation

**Figure 4:**
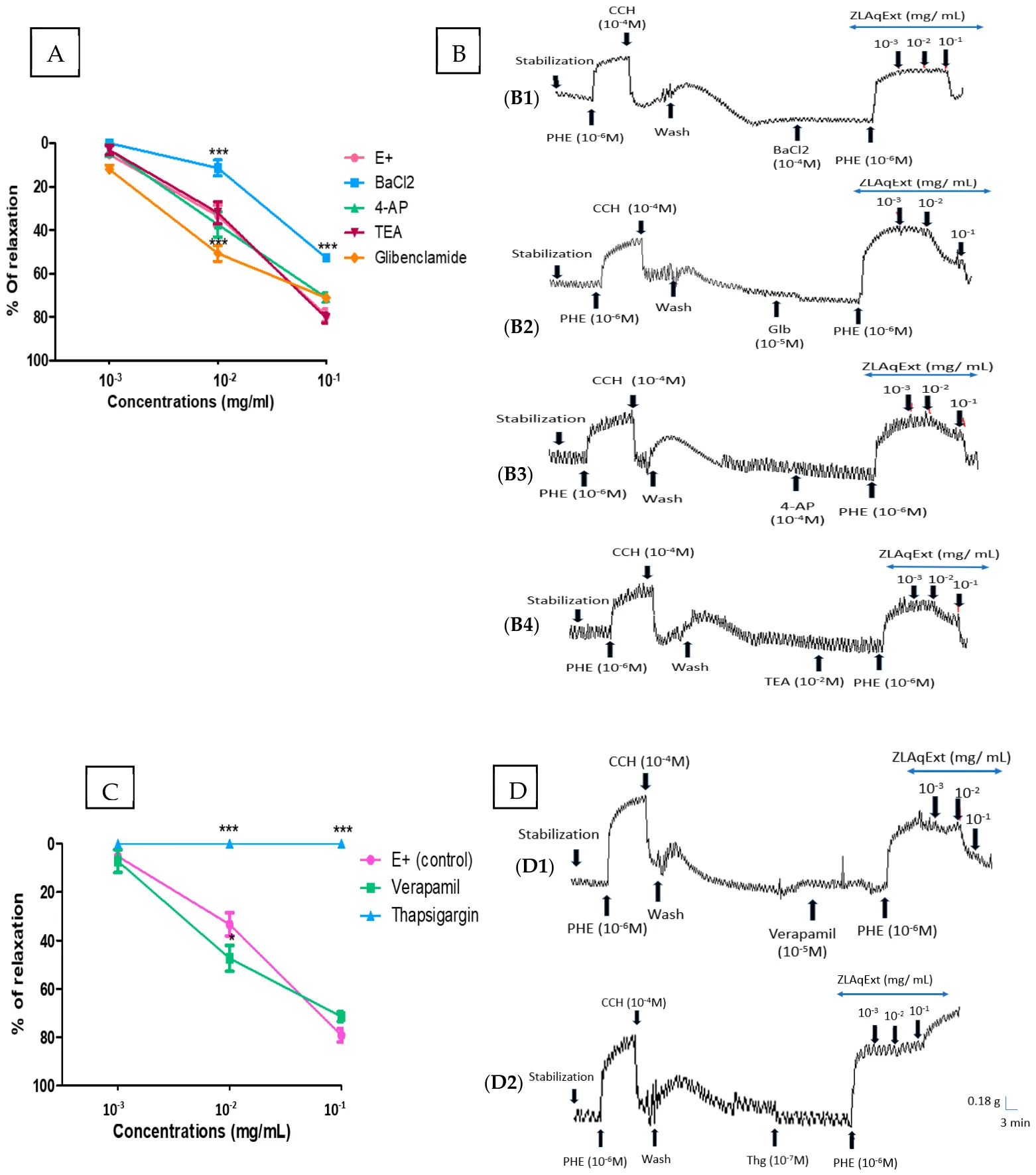
Cumulative ZLAqExt-induced relaxation in aortic rings pre-constricted with PHE (10^−6^ M) in the presence of various inhibitors. Panels (**A**,**C**): Graphs showing the effect of ZLAqExt in the presence of BaCl_2_, glibenclamide, 4-AP, TEA, verapamil, and thapsigargin. Values are expressed as mean ± SEM (*n* = 6 aortic rings/8 rats). Statistical significance was determined using two-way ANOVA with Bonferroni’s post hoc analysis; *** *p* < 0.001, * *p* < 0.05 vs. control. Panels (**B**,**D**): Representative original recordings showing the vascular effect of ZLAqExt following incubation of intact aortic rings with: (**B1**) BaCl_2_, (**B2**) glibenclamide, (**B3**) 4-AP, (**B4**) TEA, (**D1**) verapamil, (**D2**) thapsigargin. The black arrow indicates the moment of injection.

The vasorelaxant response induced by ZLAqExt was significantly attenuated following pretreatment with BaCl_2_ (Emax = 52.87 ± 1.62%; *p* < 0.001, *n* = 6) (Figure 4A,B). In contrast, no significant changes were observed after the application of 4-AP (Emax = 71.19 ± 2.15%), TEA (Emax = 80.52 ± 2.29%), or glibenclamide (Emax = 71.20 ± 1.06%).

#### 2.1.5. Involvement of VOC Channels and the SERCA Pump in ZLAqExt-Induced Vasorelaxation

The vasorelaxant activity of ZLAqExt was completely suppressed following the pretreatment with thapsigargin, a SERCA pump inhibitor (Figure 4C,D). In contrast, upon inhibition of VOC channels by verapamil, the relaxant effect remained largely unchanged, with a maximal response of 71.53 ± 2.02% (*p* > 0.05, *n* = 6).

### 2.2. Safety Profile

No mortality, clinical signs of toxicity, or behavioral changes were observed in mice treated with ZLAqExt compared to the control group receiving tap water. Furthermore, no evidence of organ hypertrophy, tissue inflammation, mucosal erosion, or significant body weight loss was recorded during the observation period. Based on these results, ZLAqExt is considered non-toxic, with a median lethal dose (LD_50_) exceeding 2000 mg/kg BW.

### 2.3. Antioxidant Properties

**Figure 5:**
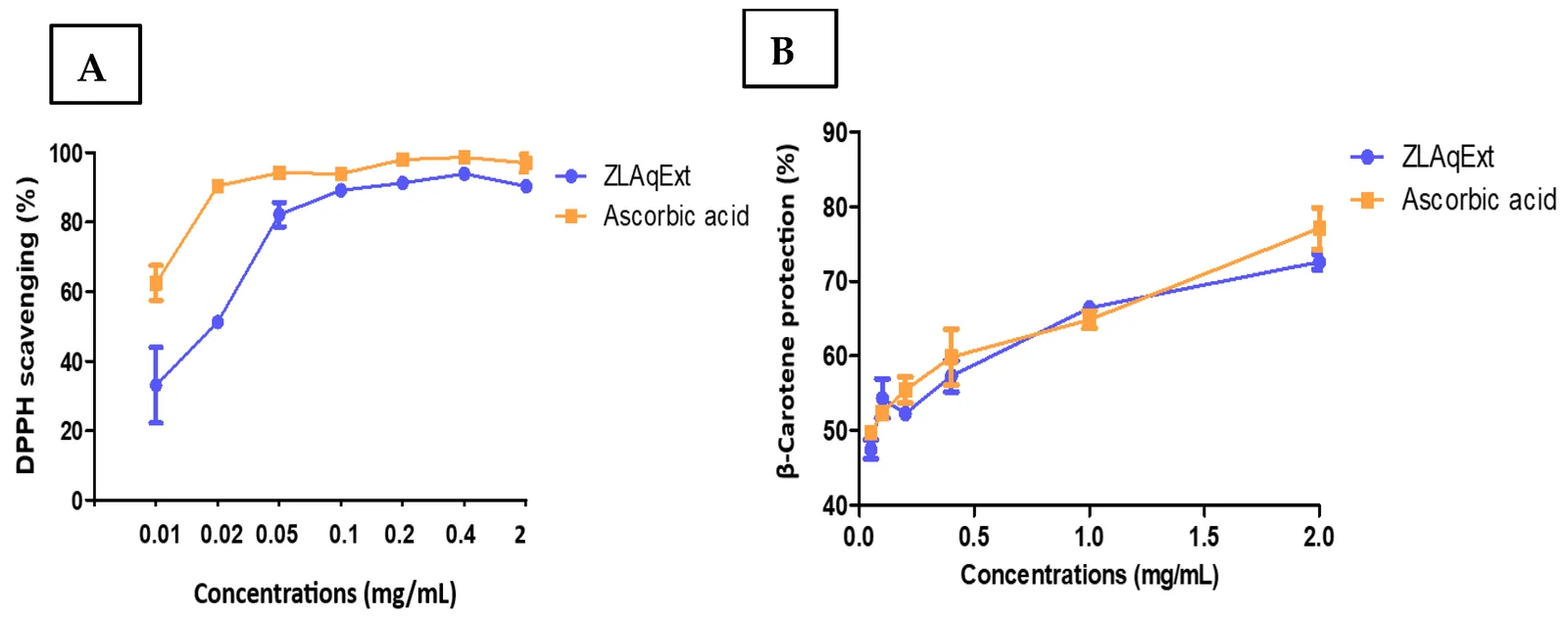
Antioxidant performance of ZLAqExt and ascorbic acid evaluated through (**A**) DPPH free radical neutralization activity and (**B**) inhibition of β-carotene oxidative discoloration.

ZLAqExt demonstrated concentration-dependent inhibitory activity against DPPH free radicals (Figure 5A). The highest scavenging effect was observed at 0.4 mg/mL, with a maximum value of 93.94%. The median inhibitory concentration (IC_50_) of ZLAqExt was determined to be 18.12 ± 1.49 µg/mL. For ascorbic acid, its activity was significant, characterized by an Emax of 98.71% and an IC_50_ value of 7.73 ± 0.83 µg/mL.

The inhibition of linoleic acid-induced β-carotene oxidation presented comparable efficacy in ZLAqExt (Emax = 72.60 ± 1.06%) and the standard ascorbic acid (Emax = 77.09 ± 2.78%) (Figure 5B). The IC_50_ value of the extract in this assay was 8.3 ± 0.014 µg/mL.

### 2.4. Phytochemical Profiling

#### 2.4.1. Quantification of Total Phenolic and Tannin Constituents

**Figure 6:**
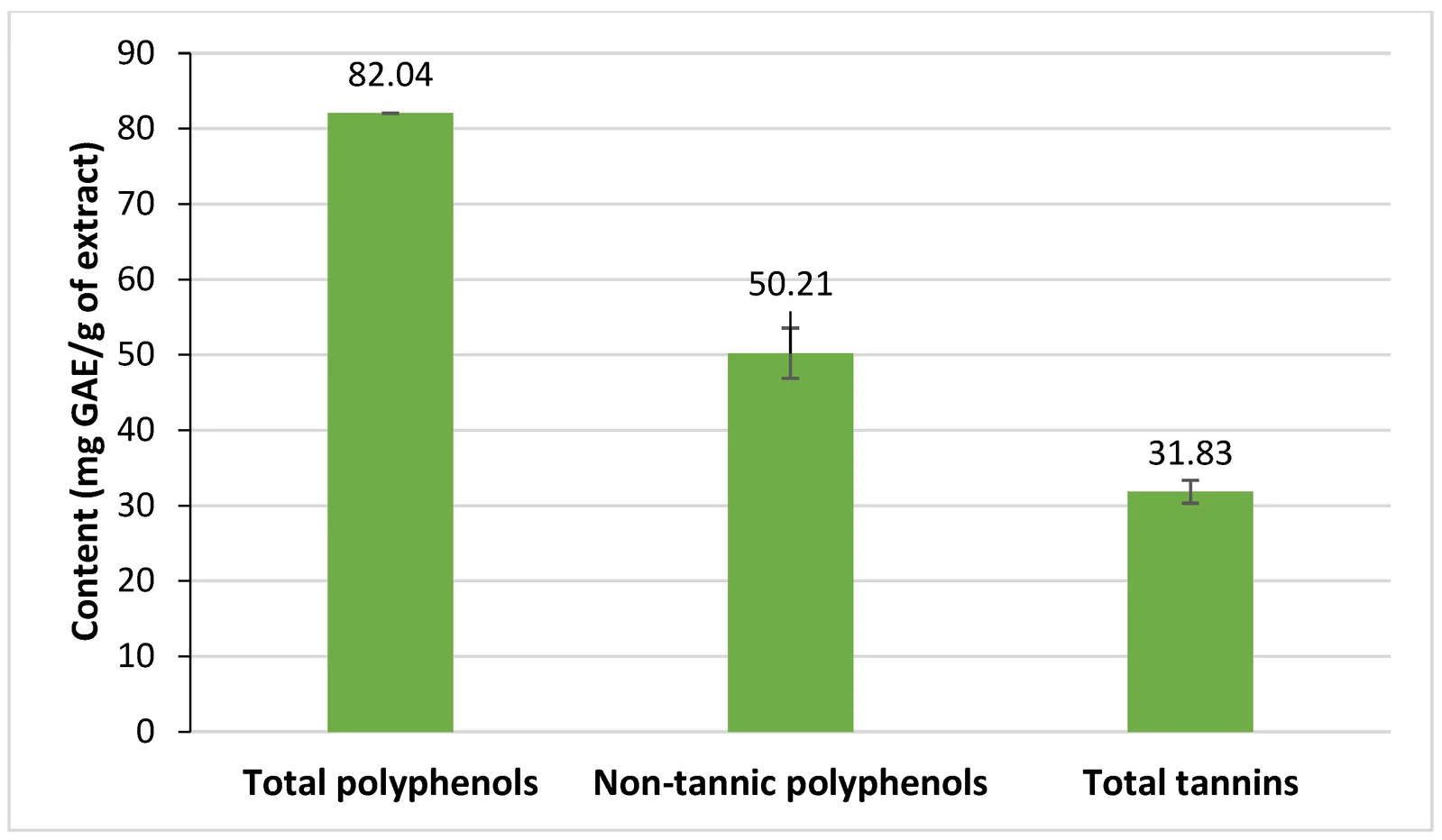
Total polyphenol and tannin contents in ZLAqExt.

ZLAqExt contained significant amounts of total polyphenols and tannins (Figure 6), with estimated contents of 82.04 ± 4.86 mg GAE/g Ext and 31.83 ± 1.53 mg GAE/g Ext, respectively. These results clearly show that, within the overall total polyphenol content, 50.21 ± 3.33 mg GAE/g Ext corresponds to non-tannin compounds. It can therefore be inferred that the aqueous leaf extract of *Z. lotus* (L.) Lam. contains more non-tannin phenolic compounds than tannins.

#### 2.4.2. UHPLC-ESI-MS Profile of ZLAqExt

**Figure 7:**
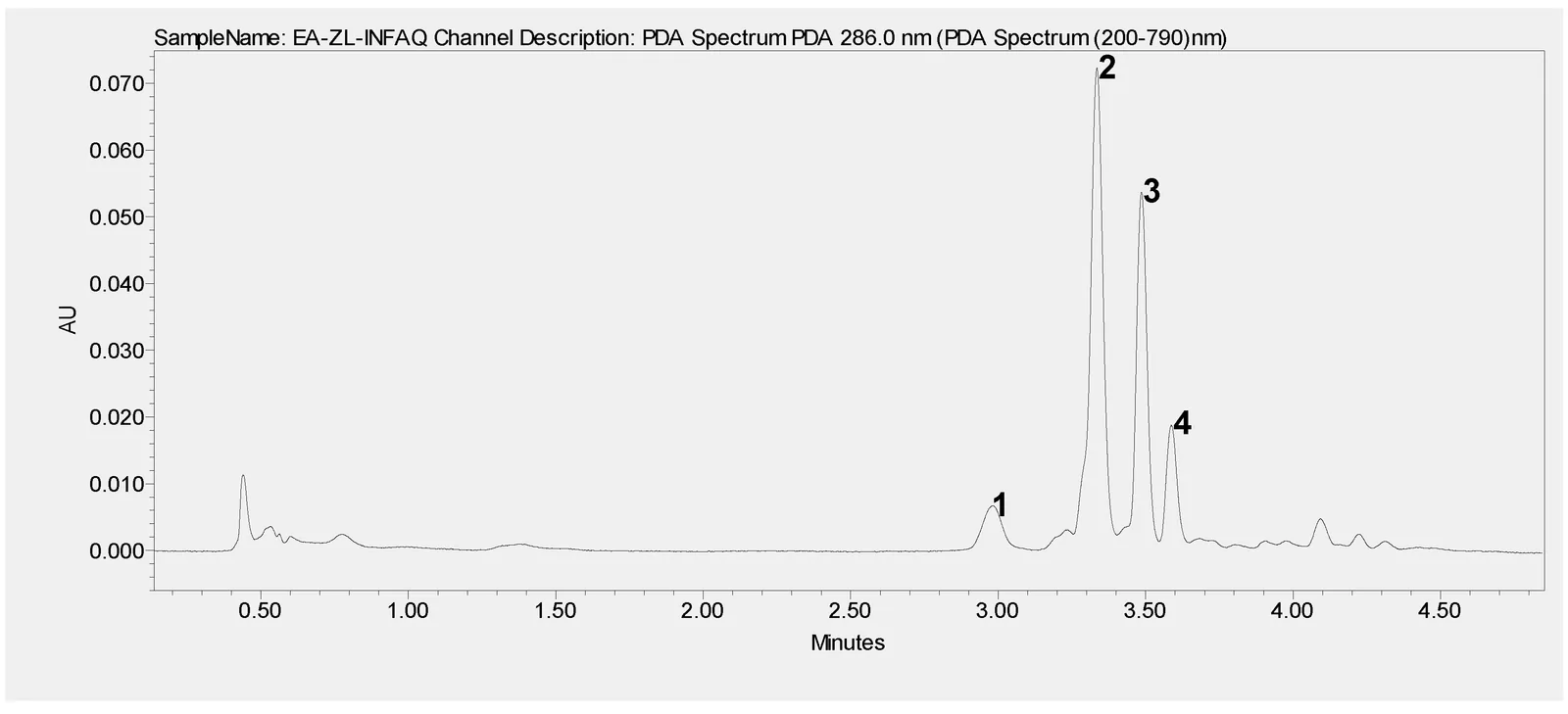
Ultra-high-performance liquid chromatography (UHPLC) chromatogram of ZLAqExt monitored at 286 nm. Peak (1): myricetin-3-O-rutinoside; peak (2): rutin; peak (3): 3′,5′-di-C-β-glucopyranosyl phloretin; peak (4): nicotiflorin.

The UHPLC-ESI-MS chromatogram and chemical composition of ZLAqExt are presented in Figure 7 and Table 1, respectively. The peaks were identified and assigned based on the matching of retention times and mass spectral data with those reported in the literature. Finally, the standards of the identified compounds were injected under the same conditions. The results indicated that our extract predominantly contained rutin (major peak), along with 3′,5′-di-C-β-glucopyranosyl phloretin, myricetin-3-O-rutinoside, and kaempferol-3-O-rutinoside (nicotiflorin).

### 2.5. Molecular Docking Analysis

To explore the potential molecular interactions underlying the vasorelaxant activity of ZLAqExt, the major phytoconstituents identified in the extract were computationally evaluated against the key proteins involved in the NO/GC/SERCA signaling pathway. This pathway plays a central role in vascular relaxation through the coordinated regulation of nitric oxide signaling, cyclic GMP production, and intracellular calcium homeostasis. Endothelial nitric oxide synthase (eNOS) was not included in the present docking analysis due to its highly complex activation mechanisms involving multiple signaling cascades and regulatory protein interactions [19].

Prior to virtual screening, the docking protocol was validated using experimentally resolved holo-structures of guanylate cyclase (GC) to assess the reliability of the predicted ligand-binding poses. As reference compounds, riociguat and CDN1163 were used as benchmark ligands for GC and SERCA, respectively. CDN1163 has been previously reported as a SERCA-binding ligand associated with modulation of Ca^2+^ homeostasis [20]. Because no co-crystallized agonist was available for the SERCA structure, blind docking was performed for this receptor.

**Figure 8:**
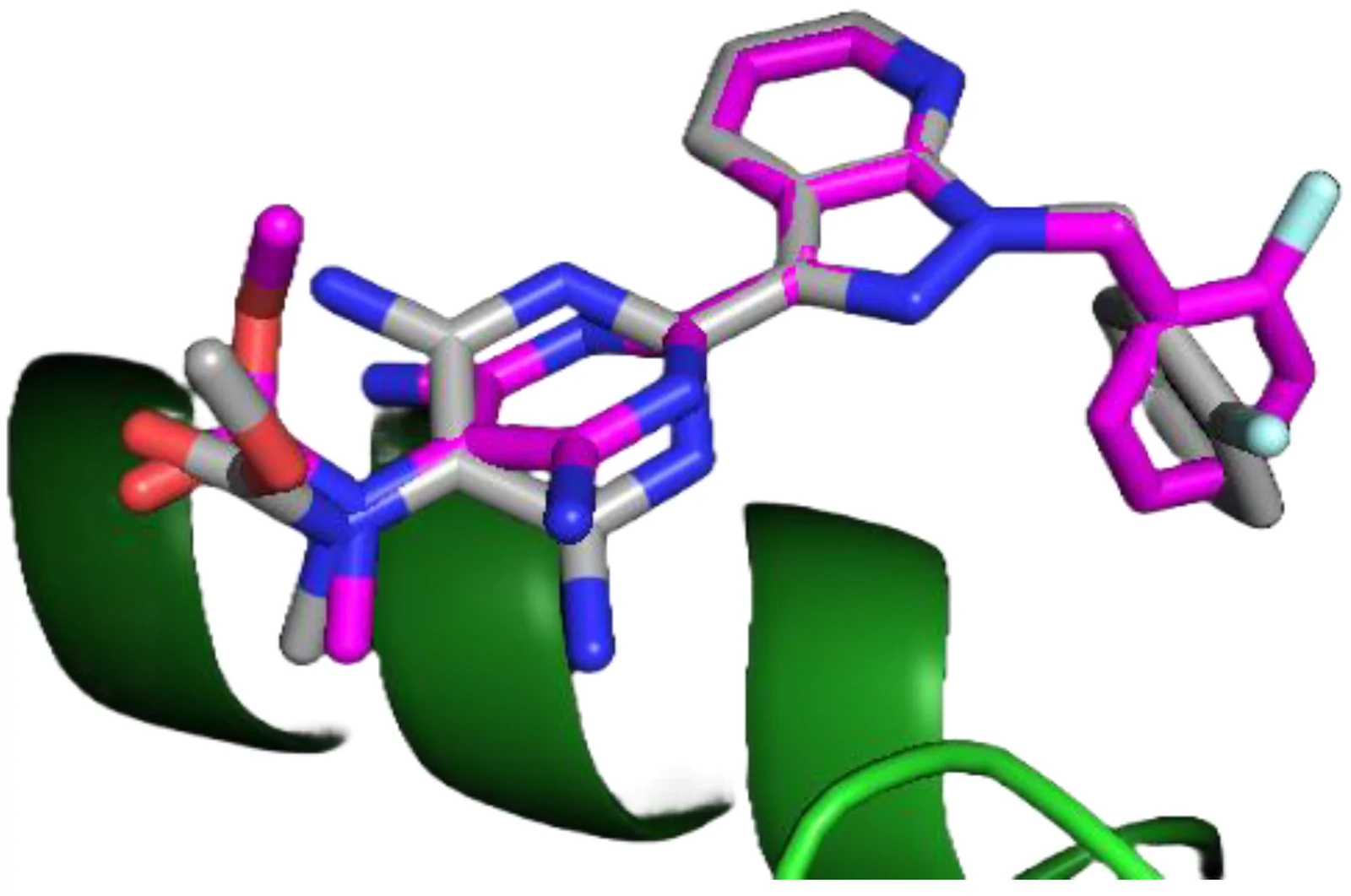
Validation (RMSD < 2.0 Å) of reverse diffusion docking for guanylate cyclase. The protein is shown as a ribbon diagram, and ligands are depicted as sticks (colored by atomic composition). X-ray ligand conformation is in gray; predicted pose is in magenta. Hydrogens are omitted for clarity.

Docking accuracy was evaluated by comparing the experimental and predicted binding conformations of the co-crystallized GC ligand, yielding an RMSD below 2.0 Å. This level of agreement supports the reliability of the predicted binding poses generated by the DiffDock framework, as illustrated in Figure 8. Following docking validation, MM-PB(GB)SA rescoring was performed to estimate ligand–receptor binding free energies and improve the reliability of affinity prediction.

**Figure 9:**
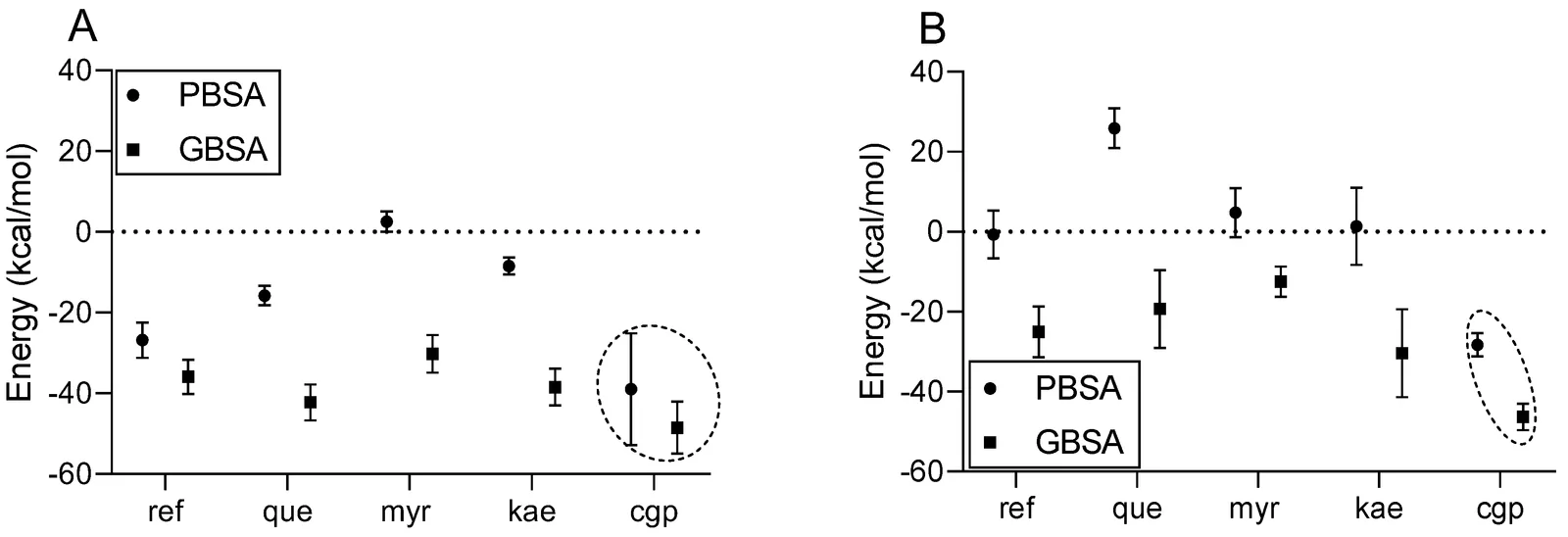
Outcomes generated from molecular docking assessments focused upon guanylate cyclase (**A**) and sarco-endoplasmic reticulum calcium^2+^-adenosine triphosphatase (**B**) protein receptor targets. The energetic demarcation threshold appears signified via a broken horizontal line indicator. The presented data corresponds to mean values accompanied by standard deviation calculations arising from implementation of PBSA (*n* = 2) combined with GBSA (*n* = 6) energetic scoring functions. The dashed circles indicate the complexes with the highest protein–ligand affinity.

Among the investigated phytoconstituents, 3′,5′-di-C-β-glucopyranosyl phloretin (CGP) exhibited the most favorable predicted binding free energies toward both GC and SERCA compared with the other tested compounds, including the reference ligand CDN1163 (Figure 9A,B). Interaction analysis further suggested that CGP established multiple stabilizing interactions within the binding pockets of both targets, including hydrogen bonding and hydrophobic contacts with key amino acid residues. The high density of hydroxyl groups present in the CGP structure may contribute to enhanced polar interactions and stabilization of the ligand–protein complexes.

Although these computational findings provide valuable structural insights into the potential interactions between ZLAqExt phytoconstituents and vasorelaxation-associated targets, further molecular dynamics simulations and experimental biophysical validation are required to confirm the stability and functional relevance of the predicted complexes.

### 2.6. Molecular Dynamics Analysis

To further evaluate the structural stability and dynamic behavior of the SERCA–ligand complexes predicted by docking, 200 ns molecular dynamics (MD) simulations were performed in triplicate for the reference SERCA system (REF-SERCA) and the complex formed with 3′,5′-di-C-β-glucopyranosyl phloretin (CGP-SERCA). MD simulations were performed exclusively on SERCA because the guanylate cyclase structure contained a co-crystallized ligand that enabled docking validation (RMSD < 2.0 Å), whereas SERCA lacked a co-crystallized reference ligand, requiring MD-based validation. This step was particularly important considering the conformational flexibility of CGP.

**Figure 10:**
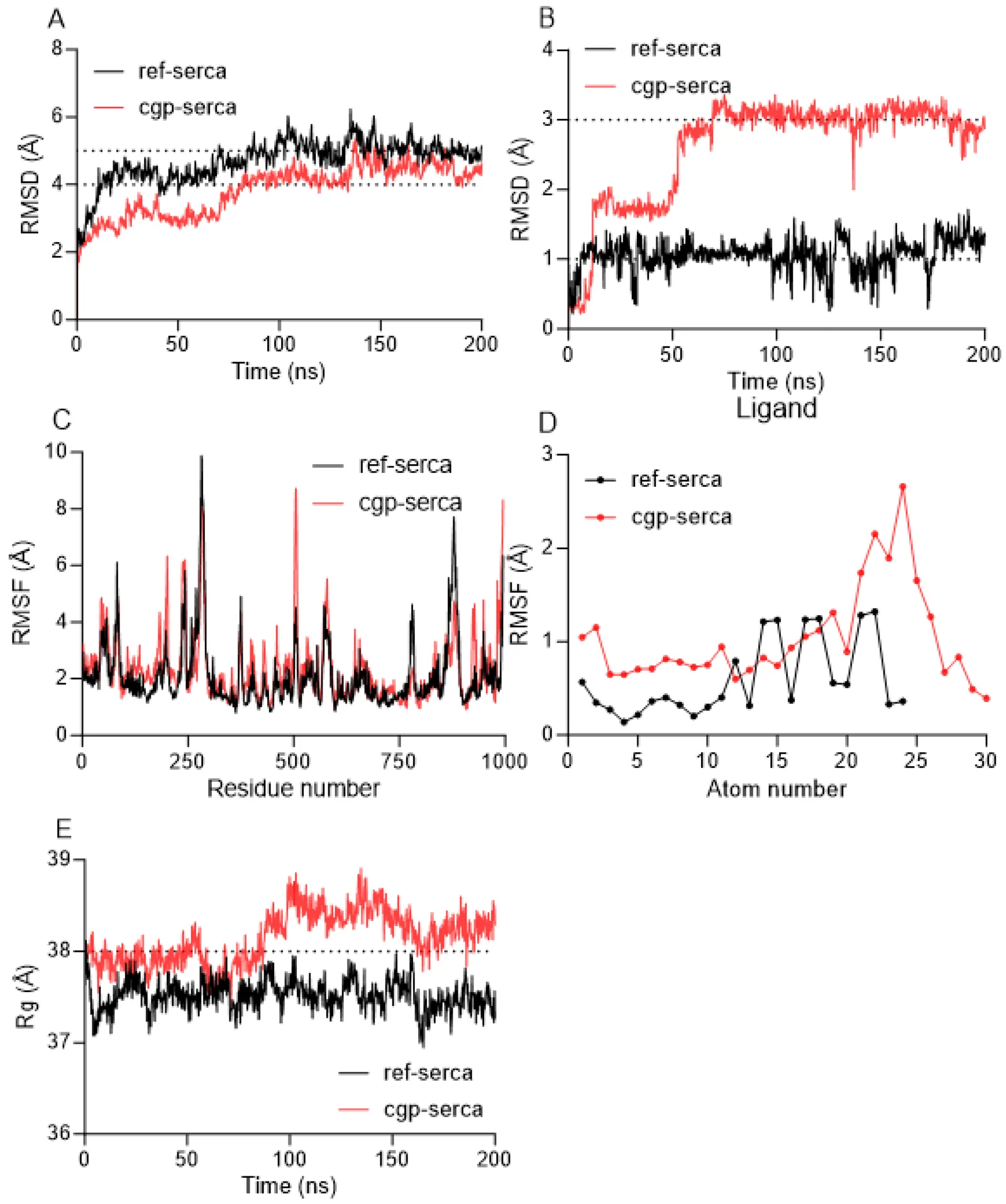
Molecular dynamics analysis of REF-SERCA and CGP-SERCA complexes over 200 ns. (**A**) Protein backbone RMSD, (**B**) ligand RMSD, (**C**) protein RMSF, (**D**) ligand RMSF, and (**E**) protein radius of gyration (Rg). The thresholds are shown as dashed lines.

Protein backbone root-mean-square deviation (RMSD) profiles (Figure 10A) showed that both systems reached equilibrium within the first ~50 ns. The REF-SERCA trajectory stabilized around ~4.5–5.0 Å, whereas the CGP-SERCA complex exhibited a slightly lower and more stable plateau (~3.5–4.5 Å), indicating that CGP binding does not destabilize the overall protein fold and may modestly enhance conformational rigidity.

Ligand RMSD (Figure 10B) revealed a clear difference in binding-mode stability. The reference ligand remained tightly confined (RMSD mostly <1.5 Å after the initial equilibration phase). In contrast, CGP displayed higher mobility, with an initial rise to ~3 Å followed by sustained fluctuations between approximately 2.5–3.2 Å, consistent with a somewhat more flexible yet persistently bound pose within the SERCA binding region.

Residue-level root-mean-square fluctuation (RMSF) analysis of the protein (Figure 10C) indicated broadly similar flexibility patterns between the two systems. Most secondary-structure elements retained low fluctuations (<2–3 Å), while loop regions and terminal segments showed the expected higher mobility. No major CGP-induced local destabilization was observed across the transmembrane domains.

Ligand atomic RMSF (Figure 10D) further illustrated the greater conformational plasticity of CGP relative to the reference compound. Several atoms of CGP exhibited elevated fluctuations (up to ~2.5 Å), reflecting the flexible glycosidic linkages, while the core scaffold remained comparatively constrained.

Radius-of-gyration (Rg) trajectories (Figure 10E) confirmed the compact and stable tertiary structure of SERCA throughout the simulations. Both systems maintained Rg values within a narrow range (~37.5–38.5 Å), with the CGP-SERCA complex showing a marginally higher average Rg that remained constant after equilibration, supporting the absence of unfolding upon ligand binding.

MM-GBSA analysis further supported CGP binding to SERCA. The calculated binding free energies were −28.82 ± 3.02 kcal/mol for REF-SERCA and −29.20 ± 5.45 kcal/mol for CGP-SERCA, indicating a slightly more favorable predicted binding energy for CGP. The CGP interaction was primarily driven by favorable van der Waals (−55.95 kcal/mol) and nonpolar solvation (−8.71 kcal/mol) contributions. Overall, these results are consistent with the molecular docking data, although the small energy difference requires cautious interpretation.

## 3. Discussion

In traditional Moroccan medicine, *Z. lotus* (L.) Lam. is used for hypertension management [12]. This study evaluated the vasorelaxant effects, phytochemical composition, and antioxidant activity of its leaf aqueous extract (ZLAqExt). Results showed no acute toxicity in mice, and experiments in isolated rat aorta indicated endothelium-dependent vasorelaxation. The primary mechanisms involve NO pathway activation and SERCA pump stimulation, with partial contributions from COX metabolites, EDHF, and inward-rectifier K^+^ channels.

ZLAqExt (10^−3^–10^−1^ mg/mL) induced concentration-dependent relaxation of phenylephrine-precontracted aortic rings with functional endothelium (EC_50_ = 0.036 ± 0.012 mg/mL; Emax = 79.25 ± 2.77%). The concentration of 10^−1^ mg/mL produced the greatest vasorelaxation, surpassing the effects reported for the aqueous extract of *Garcinia cowa* Roxb. ex Choisy leaves [21]. Furthermore, ZLAqExt demonstrated significantly greater effectiveness than extracts of *Chenopodium ambrosioides* L. [22] and *Artemisia campestris* L. [23], which produced only 13.4% and 15% relaxation at 10^−1^ mg/mL, respectively, under the same experimental protocol.

Carbachol is widely used to evaluate endothelium-dependent vascular relaxation. It activates muscarinic receptors on endothelial cells, triggering NO release. NO then diffuses into vascular smooth muscle cells, where it activates soluble guanylate cyclase (sGC), resulting in increased cGMP levels and vascular relaxation [24]. This well-characterized signaling pathway provides a basis for investigating the potential mechanisms underlying ZLAqExt-induced vasorelaxation. Therefore, the present study aimed to elucidate the cellular mechanisms involved in ZLAqExt-induced relaxation by exploring both endothelium-dependent and endothelium-independent pathways.

The vascular endothelium plays a critical role in sustaining vascular tone by releasing three key vasodilators: NO, prostacyclin (PGI_2_), and EDHF [25,26]. In our study, ZLAqExt-induced relaxation was completely abolished after endothelial removal, confirming its endothelium-dependent nature.

Numerous receptors, including muscarinic receptors, can regulate NO production in endothelial cells through eNOS activation involving Ca^2+^/calmodulin signaling [27,28,29]. Our findings demonstrated that suppression of muscarinic receptors by atropine had no impact on ZLAqExt-induced vasorelaxation. However, inhibition of NOS by L-NAME, NO scavenging by hydroxocobalamin, or inhibition of soluble guanylyl cyclase by ODQ completely abolished the response. In contrast, calmidazolium produced a slight attenuation of vasorelaxation. These results suggest that ZLAqExt-induced relaxation primarily involves activation of the endothelial NO/sGC/cGMP pathway, with a possible contribution of Ca^2+^/calmodulin-dependent mechanisms.

Beyond NO, PGI_2_ (a COX product) also mediates endothelium-derived relaxation by stimulating adenylate cyclase and increasing cAMP levels [30]. Our data suggest that ZLAqExt stimulates prostanoid production, as pretreatment with the COX inhibitor indomethacin significantly reduced the vasorelaxant effects.

EDHF contributes to vascular relaxation by inducing hyperpolarization of vascular smooth muscle cells through the activation of potassium (K^+^) channels, leading to membrane hyperpolarization and reduced calcium influx, thereby promoting relaxation [31,32]. In the present study, ZLAqExt-induced relaxation was partially attenuated by high extracellular potassium (30 mM), suggesting the involvement of EDHF-mediated mechanisms. Together with the partial inhibition observed after COX blockade, these findings suggest that EDHF- and prostanoid-related pathways may contribute to the vasorelaxant response of ZLAqExt. Furthermore, pharmacological inhibition of potassium channels revealed a partial contribution of inwardly rectifying potassium (Kir) channels to the observed relaxation.

Intracellular calcium homeostasis is a key determinant of vascular smooth muscle contraction. While voltage-operated calcium (VOC) channels regulate extracellular Ca^2+^ influx, the sarcoplasmic reticulum Ca^2+^-ATPase (SERCA) pump controls Ca^2+^ sequestration into the sarcoplasmic reticulum, contributing to smooth muscle relaxation [33,34,35].

Based on our findings, the VOC inhibitor verapamil did not alter the effect of ZLAqExt, while the response was completely suppressed after pretreatment with thapsigargin, an inhibitor of the SERCA pump. This indicates that ZLAqExt-induced vasorelaxation involves both NO availability and SERCA activity, clarifying the functional hierarchy between these two mechanisms. Endothelial NO acts as a major upstream signaling mediator required to initiate the relaxation cascade. Downstream of NO release, SERCA serves as an essential functional effector responsible for sequestering cytosolic Ca^2+^ into the sarcoplasmic reticulum to achieve smooth muscle relaxation.

Rather than assuming an exclusive, obligatory NO → sGC → cGMP → PKG → SERCA linear sequence, SERCA is best interpreted as a critical downstream component functionally coupled to NO signaling. Indeed, vascular literature indicates that NO–SERCA coupling can involve both classical cGMP/PKG-dependent pathways and direct cGMP-independent redox regulation, such as S-glutathiolation of SERCA at cysteine-674 [36,37,38].

**Figure 11:**
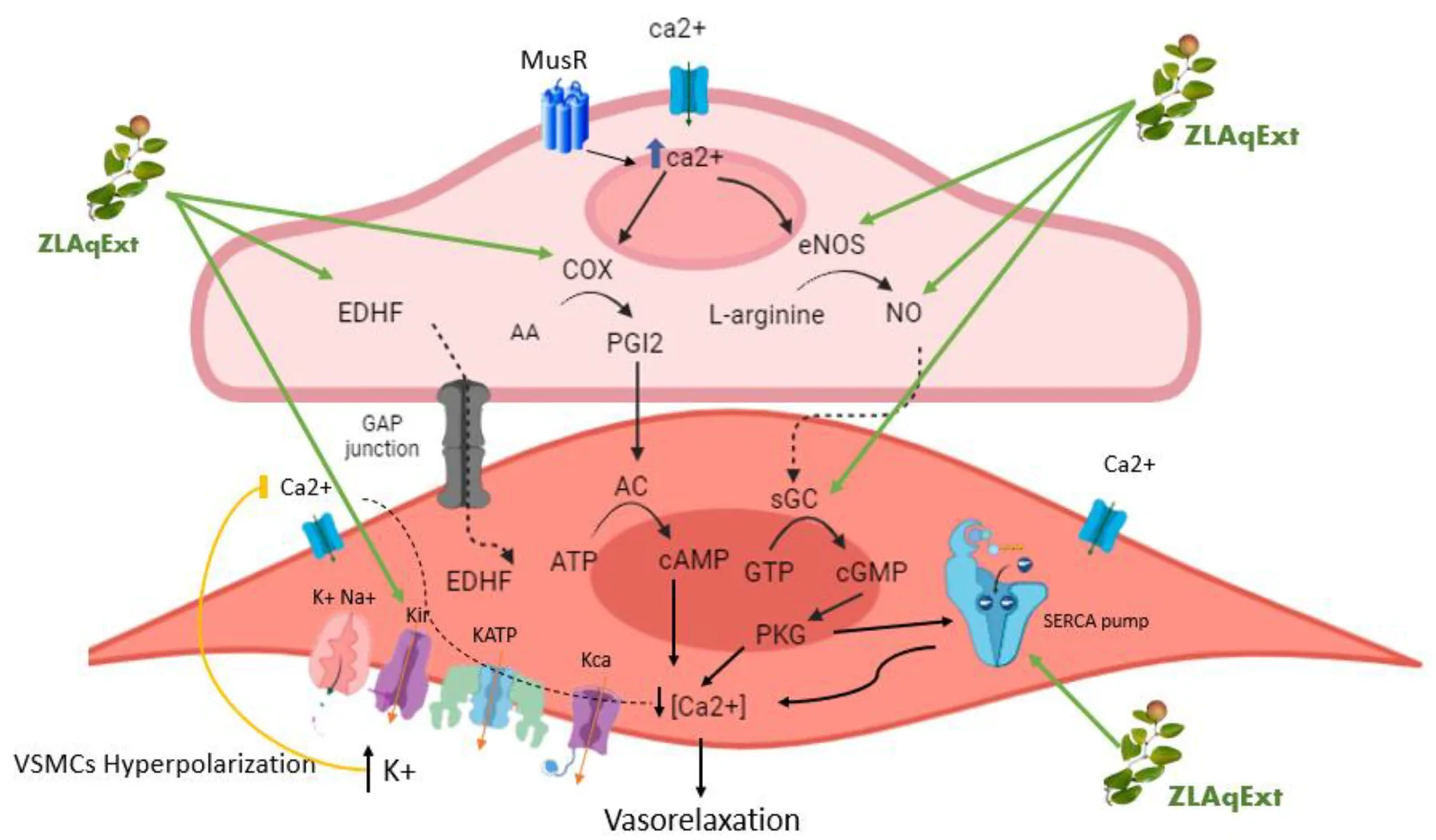
Schematic representation of the signaling pathways potentially involved in ZLAqExt’s vasodilator effect on isolated rat aortic rings.

In summary, ZLAqExt exhibits significant endothelium-dependent vasorelaxant activity. This effect primarily involves the NO pathway and SERCA pump activation, with partial contributions from COX, EDHF, and K_ir_ channel opening (Figure 11).

The oral acute toxicity of the extract was evaluated to ensure the safety of ZLAqExt. The results showed an LD_50_ higher than 2 g/kg BW, which is comparable to that reported for Algerian *Z. lotus* (L.) Lam. leaves [39]. It is well recognized that oxidative stress is closely associated with endothelial dysfunction and impaired vascular relaxation. Excessive reactive oxygen species (ROS) production can reduce NO bioavailability, thereby limiting vasodilation and increasing vascular tone [40,41].

Consequently, a complementary study was conducted to evaluate the capacity of the extract to scavenge DPPH free radicals and protect against β-carotene oxidation in vitro. ZLAqExt was characterized by a significant antioxidant potential (IC_50_ = 0.01 mg/mL), comparable to values obtained from leaves collected in Tunisia [42] and significantly more potent than the aqueous extract from Algerian leaves (IC_50_ = 0.342 mg/mL) [43].

In this context, the antioxidant activity of ZLAqExt may contribute to its vasorelaxant effects by limiting ROS-mediated impairment of endothelial function and preserving NO bioavailability. Excessive ROS production can reduce NO signaling not only through direct NO scavenging but also through oxidative pathways involving NADPH oxidase activation, peroxynitrite (ONOO^−^) formation, and tetrahydrobiopterin (BH4) oxidation, leading to eNOS uncoupling and further ROS generation [44,45]. Thus, the antioxidant properties of ZLAqExt may represent an additional mechanism contributing, at least partially, to the preservation of endothelial function and the vasorelaxant response observed in the present study. However, since vascular oxidative stress markers were not directly evaluated, this potential contribution requires further investigation.

Moreover, the present study did not include histopathological examination of vascular tissues; therefore, a direct structural protective effect of ZLAqExt on the vascular wall cannot be established. Such assessment should be considered in future studies to further investigate the potential structural effects of ZLAqExt on vascular tissues.

Phytochemical studies revealed that ZLAqExt is rich in polyphenols, particularly non-tannin compounds. UHPLC-ESI-MS analysis confirmed the presence of flavonoids, with quercetin-3-O-rutinoside (rutin) identified as the major compound, followed by 3′,5′-di-C-β-glucopyranosylphloretin, kaempferol-3-O-rutinoside, and myricetin-3-O-rutinoside. These findings are consistent with the data reported by Rached et al. (2019) on the phytochemical profile of wild jujube from western Algeria, where leaves and fruit predominantly contained flavonoid derivatives [16]. Notably, 3′,5′-di-C-β-glucopyranosylphloretin was identified for the first time among the main flavonoids in leaves from the eastern region of Morocco. However, when the aqueous extract of leaves collected from the Sidi Sliman region of Morocco was prepared using the same extraction method (infusion), its phytochemical analysis revealed that phenolic acids were the main compounds. Gallic acid, vanillic acid, caffeic acid, pyrogallol, and chlorogenic acid were found to be the predominant compounds, along with p-hydroxybenzoic acid, p-coumaric acid, naringin, and trace amounts of rutin [46]. Furthermore, rutin was identified as the major constituent in the methanolic and water extracts of Algerian *Z. lotus* leaves [47]. Myricetin 3-O-rutinoside and kaempferol 3-O-rutinoside were also reported in leaf samples from Western Algeria [16].

There is ample evidence that the flavonoids found in ZLAqExt, especially rutin, possess potent endothelium-dependent vasorelaxant properties mediated through the NO/sGC pathway and the activation of K_ATP_ channels [48,49]. Myricetin, the aglycone of myricetin-3-O-rutinoside, has been shown to interact with sGC and promote the overexpression of SERCA [50,51], suggesting that it may also contribute to the observed vasorelaxant effects. Additionally, kaempferol-3-O-rutinoside has been reported to elicit endothelium-independent relaxation (Emax = 76%) in isolated rat aorta via a cholinergic pathway [52].

Considering the presence of multiple bioactive flavonoids in ZLAqExt, including rutin, myricetin-3-O-rutinoside, 3′,5′-di-C-β-glucopyranosylphloretin, and kaempferol-3-O-rutinoside, the observed biological activity may result from the contribution of individual constituents as well as from potential synergistic or additive interactions among these compounds within the extract matrix [53]. Therefore, the pharmacological effects of the aqueous extract should be interpreted in the context of its complex phytochemical composition rather than being attributed solely to a single constituent. Further studies using purified compounds and combination assays are required to elucidate the specific contribution and possible interactions among these flavonoids.

The translation of an ex vivo effective vasorelaxant concentration into systemic pharmacological effects requires consideration of pharmacokinetic parameters and tissue exposure [54]. In the present study, ZLAqExt demonstrated a potent vasorelaxant response in isolated aortic rings at an effective concentration of 0.01 mg/mL, highlighting its intrinsic activity in the vascular preparation. In agreement with these findings, our previous in vivo investigation in normotensive rats demonstrated a maximal hypotensive effect following intravenous administration of ZLAqExt via the femoral vein at a dose of 20 mg/kg [55]. However, this administration route bypasses gastrointestinal absorption and first-pass metabolism and therefore does not provide information regarding oral bioavailability. The difference between ex vivo effective concentration and in vivo administered dose reflects the complexity of pharmacological processes, including systemic distribution, tissue exposure, metabolism, and plasma protein binding [54,56].

The major flavonoid derivatives identified in ZLAqExt are generally known to undergo intestinal absorption and extensive metabolic transformation. In particular, rutin and other flavonoid glycosides often exhibit limited systemic availability as parent compounds due to intestinal microbiota transformation and phase II metabolism, generating circulating conjugated metabolites that may contribute to their biological effects [57,58,59,60]. However, the pharmacokinetic profile, oral bioavailability, and systemic exposure of ZLAqExt constituents following oral administration remain to be established. Since intravenous administration bypasses gastrointestinal absorption and hepatic first-pass metabolism [61], further dedicated pharmacokinetic/pharmacodynamic (PK/PD) studies are required to determine the absorption profile, distribution, and potential circulating metabolites that may contribute to the vascular effects of ZLAqExt.

The MM-PB(GB)SA analysis provided additional insight into the predicted binding affinities of the investigated phytoconstituents toward the selected protein targets. Among the evaluated compounds, 3′,5′-di-C-β-glucopyranosyl phloretin (CGP) displayed the most favorable predicted binding free energies against both GC and SERCA compared with the other tested molecules, suggesting that CGP may represent one of the potential contributors to the interaction with these vascular targets. Guanylate cyclase plays a central role in NO-mediated vasodilation through cGMP generation, whereas SERCA is critically involved in intracellular calcium homeostasis in vascular smooth muscle cells [62]. Therefore, the predicted interaction profile of CGP with these targets may partially contribute to the vasorelaxant effects observed experimentally for ZLAqExt.

The favorable ΔG values obtained by MM-PB(GB)SA supported the docking predictions generated using the DiffDock framework. Interaction analysis suggested that CGP forms stabilizing interactions within the binding pockets of GC and SERCA, including hydrogen bonding and hydrophobic contacts. The presence of multiple hydroxyl groups in the CGP structure may contribute to these interactions. Nevertheless, the biological activity of the whole extract may be influenced by the combined contribution of multiple phytoconstituents, including major compounds such as rutin, rather than by CGP alone.

Taken together, the ex vivo vascular experiments and in silico findings suggest that ZLAqExt contains bioactive phytoconstituents potentially involved in vascular relaxation pathways. However, these computational predictions should be interpreted cautiously, as docking and MM-PB(GB)SA analyses alone cannot confirm functional modulation of the investigated targets. Further molecular dynamics simulations, biochemical assays, and in vivo studies are required to validate the biological relevance of these interactions.

Overall, this study provides preliminary structural insights into the possible contribution of *Z. lotus* phytoconstituents to vascular relaxation and supports further investigation of CGP, together with other major constituents such as rutin, as potential contributors to the endothelium-dependent vasorelaxant mechanisms of ZLAqExt.

## 4. Materials and Methods

### 4.1. Chemicals

The pharmacological agents employed in this study consisted of (R)-(-)-phenylephrine hydrochloride [Phe] (procured from Sigma Aldrich, Schnelldorf, Germany), 1H-[1,2,4]oxadiazolo[4,3-a]quinoxalin-1-one [ODQ] (supplied by Cayman Chemical, Ann Arbor, MI, USA), 4-aminopyridine [4-AP] (obtained from Alfa Aesar, Karlsruhe, Germany), atropine and verapamil hydrochloride (sourced respectively from Sigma Aldrich, Shanghai, China), indomethacin (acquired from Sigma Aldrich-Fluka, Milan, Italy), Nω-Nitro-L-arginine methyl ester hydrochloride [L-NAME] (provided by Sigma Aldrich, Buchs, Switzerland), hydroxocobalamin hydrochloride (supplied by Fluka, Saint Louis, MO, USA), thapsigargin (procured from Sigma Aldrich, Jerusalem, Israel), barium chloride dihydrate [BaCl_2_] (obtained from VWR, Leuven, Belgium), calmidazolium, glibenclamide, tetraethylammonium chloride hydrate [TEA], and carbamylcholine chloride [carbachol, CCH] (all procured from Sigma Aldrich, Saint Louis, MO, USA).

Stock solutions of ODQ and thapsigargin were prepared in dimethyl sulfoxide (DMSO), whereas ethanol served as the carrier for indomethacin preparation. All other supplementary reagents were dissolved in distilled water.

### 4.2. Plant Material

In April 2022, samples of *Z. lotus* (L.) Lam. leaves, locally known as “Sedra,” were gathered in the vicinity of Figuig City in southeastern Morocco (coordinates 32°06′10.6″ N 1°14′41.0″ W). Professor Mostapha ELACHOURI from the Biology Department of the Faculty of Sciences at University Mohamed First in Oujda, Morocco, processed and registered the specimen in the department’s herbarium for scientific reference under voucher number (HUMPOM428). The plant’s name was verified and validated as suggested by http://www.worldfloraonline.org.

### 4.3. Aqueous Extract Preparations

The aqueous extract was prepared based on traditional Moroccan practices. Twenty grams of crushed dried leaves were infused for 30 min in 200 mL of boiled distilled water. The aqueous extract (AqExt) was subjected to filtration, and the filtrate was concentrated in vacuo at 50 °C using a rotary evaporator (EYELA N-1300, Tokyo, Japan). The yield of the extraction was 15%. The crude extract was conserved in a plastic bottle in the freezer at −20 °C until use.

### 4.4. Experimental Animals

The present study involved male Wistar rats (average weight 300 g), and albino mice, weighing 25–35 g. The animals were bred, housed, and acclimatized to regular environmental conditions in the animal facility of the Faculty of Sciences, Oujda, Morocco. The animals were fed a specifically tailored rat chow, had unrestricted access to water, and were exposed to a regulated 12 h light–dark cycle. The recommendations, given in the “Guide for the Care and Use of Laboratory Animals” [63], were strictly followed throughout the experimental period. Ethical approval for all the experimental protocols was granted by the Faculty of Sciences, Mohamed First University, Oujda, Morocco (Authorization No. 11/2023-LBBES).

### 4.5. Preparation of Thoracic Aortic Rings

Male Wistar rats (*n* = 20) were anesthetized through intraperitoneal administration of urethane (20%, 1 mL/100 g BW) to perform a thoracotomy. The thoracic aorta was swiftly and delicately removed and was rapidly placed in cold oxygenated Krebs–Henseleit solution (composition in mM: NaCl 119, KCl 4.7, CaCl_2_ 2.6, MgSO_4_ 1.2, KH_2_PO_4_ 1.2, NaHCO_3_ 25, and glucose 11). Subsequently, the aorta was thoroughly cleaned under a binocular loupe to remove fat and connective tissue adhesions. Afterwards, the isolated aorta was segmented into rings approximately 3–4 mm in length. The rings were delicately mounted between two parallel stainless-steel hooks, which were closed in opposition to each other and suspended in an organ chamber (EMKA Technologies, Paris, France) filled with 11 mL of Krebs solution (95% O_2_/5% CO_2_, 37 °C, pH 7.4). One hook was attached to an isometric force transducer (EMKA Technologies, Paris, France), which detects the vasomotricity of the aortic rings. An amplifier (EMKA Technologies, France) directly amplified the generated voltage signal, which was subsequently acquired by usbACQ (EMKA Technologies, France) and was recorded by the data visualization and analysis software (IOX2 v2.8). The rings were stretched to a basal tension of 1 g and were left to stabilize for 30 min. Endothelial integrity was confirmed when carbachol (10^−4^ M) induced ≥60% relaxation following phenylephrine (10^−6^ M)-induced contraction. Endothelium-denuded rings were prepared by gentle mechanical abrasion of the intimal surface; denudation was verified by the absence of carbachol-induced relaxation. Before experiments, the rings were rinsed twice and re-equilibrated for 30 min. The vasorelaxant effect of ZLAqExt and its endothelium dependence were assessed using the rings with intact or denuded endothelium. After stabilization and phenylephrine-induced contraction, cumulative concentrations of ZLAqExt (10^−3^, 10^−2^, 10^−1^ mg/mL) were applied once the contractile response reached a steady state.

### 4.6. Mechanism of Action Involved in the Vascular Effect

To clarify the mechanisms underlying ZLAqExt-induced vasorelaxation, endothelium-intact aortic rings were preincubated for 20 min with various pharmacological inhibitors [23,50]: L-NAME (10^−4^ M, NOS inhibitor), ODQ (10^−5^ M, sGC inhibitor), hydroxocobalamin (3 × 10^−5^ M, NO scavenger), indomethacin (10^−5^ M, COX inhibitor), BaCl_2_ (10^−4^ M, K_ir_ blocker), 4-AP (10^−4^ M, K_v_ blocker), TEA (10^−2^ M, K_Ca_ blocker), glibenclamide (10^−5^ M, K_ATP_ blocker), calmidazolium (10^−3^ µM, Ca^2+^-CaM/eNOS binding inhibitor), KCl (30 mM, EDHF inhibitor), atropine (10^−6^ M, muscarinic antagonist), verapamil (10^−5^ M, VOC inhibitor), and thapsigargin (10^−7^ M, SERCA inhibitor). Following preincubation, the cumulative concentrations of ZLAqExt (10^−3^–10^−1^ mg/mL) were added after phenylephrine-induced contraction. Responses were compared to control conditions (ZLAqExt alone). Each ring was tested under a single condition (inhibitor or control) to preserve tissue integrity and avoid desensitization caused by repeated exposures; all experiments were performed independently on the rings from separate rats.

### 4.7. Acute Toxicity

Acute toxicity assessment was undertaken according to OECD guideline 423 [64] using the Limit Test procedure, with some experimental modifications. The study was carried out on 12 albino mice (5 females and 7 males, weighing 25–35 g), assigned to control and test groups (*n* = 6 per group). The control group received distilled water as a vehicle, whereas the test group was given ZLAqExt. The animals were fasted for 16 h before the initiation of the experiment. After weighing, they received a single dose of 2 g/kg BW of ZLAqExt via gastric intubation (10 mL/kg). The mice were subjected to continuous individual observation for 30 min immediately after administration, followed by 24 h, with specific emphasis on the first 4 h. This monitoring continued for 14 days, enabling the recording of any instances of mortality or manifestations of general signs and symptoms of toxicity. Upon completion of treatment, the surviving animals were euthanized. Subsequently, a macroscopic examination was conducted on the internal organs, including the heart, lungs, liver, and kidneys.

### 4.8. DPPH Free Radical Neutralization Assay

One milliliter of a 0.1 mM 2,2-diphenyl-1-picrylhydrazyl (DPPH) solution prepared in methanol was combined with an identical 1 mL volume of the extract dilutions at concentrations of 5, 10, 20, 50, 100, 200, 400, and 2000 µg/mL. All the dilutions were previously dissolved in methanol, according to de la Rosa et al. (2011) [65], incorporating selected adjustments. Ascorbic acid served as the positive control and was evaluated at equivalent concentrations to ZLAqExt. Pure methanol provided the blank reference. Triplicate preparations were performed for every concentration examined. The reaction mixtures underwent a 30 min incubation phase at room temperature, shielded from light. Upon completion of incubation time, the absorbance was measured at 515 nm. An extract blank was prepared for each ZLAqExt concentration, and its absorbance at 515 nm was subtracted from the corresponding sample absorbance to correct for the intrinsic absorbance of the extract. The percentage of DPPH free radicals neutralized by the extract was calculated employing the formula:

$$Neutralisation\;(\%)=\frac{Ac-At}{Ac}\times 100$$

wherein Ac designates the absorbance of the control and At signifies the absorbance of the test sample.

### 4.9. β-Carotene Oxidation Protection Assay

An emulsified formulation comprising β-carotene and linoleic acid was prepared, following the method of Bekkouch et al. (2019) [66], albeit with certain procedural variations. Briefly, 10 mL of β-carotene solution (solubilized in chloroform at 0.2 mg/mL) was combined with 20 mg of linoleic acid and 200 mg of Tween 40. Following vigorous mixing, the chloroform was removed under vacuum at 40 °C. Subsequently, 100 mL of distilled water was incorporated under robust agitation to form the emulsion. Serial dilutions of ZLAqExt (50, 100, 200, 400, 1000, and 2000 µg/mL) and the standard ascorbic acid solution were prepared in methanol. An extract blank was prepared for each ZLAqExt concentration, and its absorbance at 490 nm was subtracted from the corresponding sample absorbance to correct for the intrinsic absorbance of the extract. A volume of 200 µL of either the extract or the reference was added to 5 mL of the emulsion. The reaction mixtures were incubated in a water bath at 50 °C for 2 h. The absorbance was measured at 490 nm immediately upon addition of the emulsion (A0) and following 120 min of incubation (At).

The degree of β-carotene preservation was determined using the expression:

$$\beta \text{-}carotene\;preservation\;\left(\%P\right)=\;\frac{At}{A0\;}\times 100$$

where At and A0 represent the absorbance values at 120 min and 0 min, respectively.

### 4.10. Polyphenolic Constituents’ Quantification

The Folin–Ciocalteu colorimetric assay was employed to determine the total phenolic content of ZLAqExt, as described for 96-well microplates [67]. Briefly, a 20 µL aliquot of the extract (3.33 mg/mL) was transferred to each well and mixed with 100 µL of Folin–Ciocalteu reagent (10%). Thereafter, 80 µL of sodium carbonate solution (106 g/L) was added to the mixture. Following 5 min of orbital shaking, the absorbance was measured at 760 nm using a SPECTROstar Nano spectrophotometer (BMG LABTECH, Ortenberg, Germany), with measurements recorded every 15 min for 90 min. The negative control followed the same procedure, substituting the extract with 20 µL of distilled water. The phenolic concentration was calculated from a gallic acid standard curve (0, 6.25, 12.5, 25, 50, and 100 mg/mL) and is presented as milligrams of gallic acid equivalents per gram of dry extract (mg GAE/g).

### 4.11. Tannic Constituents’ Quantification

Total tannin content in ZLAqExt was measured using the hide-powder method [67], which involves adsorbing tannins with hide powder and quantifying the resulting polyphenol difference. A 500 µL aliquot of the extract (3.33 mg/mL) was incubated with 10 mg of non-chromated hide powder for 1 h, and then centrifuged (4350 rpm, 13 min). The polyphenols in the supernatant were determined following the procedure mentioned in Section 4.10. Total tannin content was calculated as the difference between initial and post-adsorption polyphenol levels and expressed as mg GAE/g.

### 4.12. UHPLC-ESI-MS Phytochemical Profiling

An Acquity UPLC H-Class system (Waters, Guyancourt, France) equipped with a photodiode array detector (PDA) coupled to a QDa ESI-quadrupole mass spectrometer (Waters Corporation, Milford, MA, USA) was employed. Data acquisition, instrument control, and analysis were managed using Empower 3 software. Chromatographic separation was performed using a multi-step elution gradient: 10–80% solvent B (0–9 min), followed by an increase to 100% B (9.01–10.49 min), and returning to 10% B (11.5–14 min) for system re-equilibration. The binary mobile phase system comprised 0.1% (*v*/*v*) aqueous formic acid (Phase A) and acetonitrile supplemented with 0.1% formic acid (Phase B). Samples (2 µL), dissolved at 1 mg/mL in analytical-grade MeOH and filtered through a 0.4 µm PTFE membrane, were injected for analysis.

### 4.13. Molecular Docking

The 3D structures of the candidate ligands investigated in this study included quercetin 3-O-rutinoside (QUE, PubChem ID: 5280805), myricetin-3-O-rutinoside (MYR, PubChem ID: 21577860), and kaempferol 3-O-rutinoside (KAE, PubChem ID: 5318767). These structures were obtained from the PubChem chemical repository https://pubchem.ncbi.nlm.nih.gov/. while the configuration for 3′,5′-di-C-β-glucopyranosyl phloretin (CGP), which was unlisted in the PubChem archive, was constructed de novo using MarvinSketch 21.2 software. Regarding the receptor targets, the 3D protein structures for guanylate cyclase (GC, PDB ID: 7D9R) and sarco-endoplasmic reticulum calcium^2+^-ATPase (SERCA, PDB ID: 4H1W) were retrieved from the RCSB Protein Data Bank https://www.rcsb.org/.

Protein structures were prepared by removing crystallographic water molecules except those involved in ligand stabilization. Hydrogen atoms and Kollman/AMBER charges were added, and protonation states of ionizable residues were assigned at physiological pH. Energy minimization was subsequently performed to relieve steric clashes prior to docking. To further improve the structural quality, receptor models were refined using the SWISS-MODEL server to correct structural gaps and optimize the local geometry. Stereochemical validation of the refined protein structures was subsequently performed using the PROCHECK program according to previously established protocols [68,69].

Initial ligand binding poses were generated using the DiffDock 1.0 framework, a diffusion-based deep learning approach for protein–ligand pose prediction [70]. Reference ligands, including riociguat for GC and CDN1163 for SERCA, were used as control compounds to benchmark the docking performance. A DiffDock 1.0 confidence score (DDS) greater than 0 was used to identify the most reliable binding poses. Docking protocol validation for GC was achieved through redocking of the co-crystallized ligand, yielding a root mean square deviation (RMSD) value below 2.0 Å between experimental and predicted poses. Because no co-crystallized agonist was available for the SERCA structure, a site-agnostic (blind) pose prediction was performed for this receptor using the DiffDock framework, which does not require a pre-defined search grid.

To improve the reliability of binding affinity estimation, docking poses were subsequently refined and rescored using the MM-PB(GB)SA (molecular mechanics/Poisson–Boltzmann/generalized Born surface area) to implicit solvation approach implemented in the fastDRH server [71]. Ligand–receptor binding free energies were expressed as ΔGpbsa and ΔGgbsa values.

### 4.14. Molecular Dynamics Simulations

All molecular dynamics (MD) simulations were carried out using the AMBER 22 software package. The protein backbone and side chains were represented by the FF99SB force field, whereas ligand parameters were derived from the Generalized Amber Force Field (GAFF) as described by J. Wang and colleagues (2004) [72]. Partial atomic charges for the ligands were obtained via the AM1-BCC charge model, utilizing the antechamber tool within AmberTools, following standard procedures reported in the literature [73]. Each solvated system was placed in a cubic periodic box filled with TIP3P water molecules, and overall electroneutrality was achieved by adding sodium (Na^+^) counterions through the tLEap module. Long-range electrostatic interactions were handled using the particle-mesh Ewald (PME) technique beyond the specified cutoff, while hydrogen-involving bond lengths were constrained with the SHAKE algorithm [74,75] permitting a time step of 2.0 fs. Temperature was regulated at 310 K via a Langevin thermostat. Prior to the production phase, each system underwent energy minimization consisting of 10,000 steps, followed by equilibration under NVT (1.0 ns) and NPT (1.0 ns) conditions. Subsequently, a 200 ns production run was performed for every system. Post-simulation, binding free energies were computed using a Python 3.11.6-based MM-GBSA approach, incorporating implicit solvent model according to well-established protocols [76].

### 4.15. Statistics

Data is reported as mean ± SEM, where n is the number of independent experiments. Results were analysed with GraphPad Prism 5 using one-way and two-way ANOVA, followed by Dunnett’s or Bonferroni’s post hoc tests. Statistical significance was established at *p* < 0.05.

## 5. Conclusions

This study provides the first comprehensive demonstration that the aqueous leaf extract of *Ziziphus lotus* induces significant, endothelium-dependent vasorelaxation in isolated rat aortic rings. Mechanistic investigations using pharmacological inhibitors establish that this effect is principally mediated through the activation of the NO/sGC/cGMP signaling pathway, coupled with stimulation of SERCA pumps. Partial contributions from COX-derived prostacyclin, EDHF, and K_ir_ channels were also identified. The aqueous extract demonstrated a favorable acute safety profile in mice (LD_50_ > 2 g/kg BW) and potent antioxidant activity, which may contribute to the preservation of endothelial function and NO bioavailability. UHPLC-ESI-MS profiling revealed a flavonoid-rich composition, with rutin as the predominant compound. Notably, AI-based molecular docking combined with MM-PB(GB)SA scoring identified 3′,5′-di-C-β-glucopyranosyl phloretin as a high-affinity dual ligand for both GC and SERCA, suggesting that it may represent one of the contributors to the observed vasorelaxant effects. However, the vascular activity of ZLAqExt is likely the result of the combined contribution and possible interactions among multiple phytoconstituents, including major compounds such as rutin. Collectively, these findings support the traditional Moroccan use of *Z. lotus* leaves for hypertension management and position ZLAqExt as a promising natural therapeutic candidate for vascular dysfunction, warranting further in vivo efficacy studies and investigations using isolated compounds to determine the specific contribution of individual constituents and possible interactions responsible for its vasorelaxant activity.

## Figures and Tables

**Table 1:** Ultra high-performance liquid chromatography (UHPLC) and electrospray ionization–mass spectrometry (ESI-MS) negative ion analysis of aqueous extract compounds of *Z. lotus* leaves.

Peak	RT (min)	*m*/*z* [M-H]^−^	Compound	Reference	Chemical Structure
1	2.980	625.34	Myricetin 3-O-rutinoside	[16]	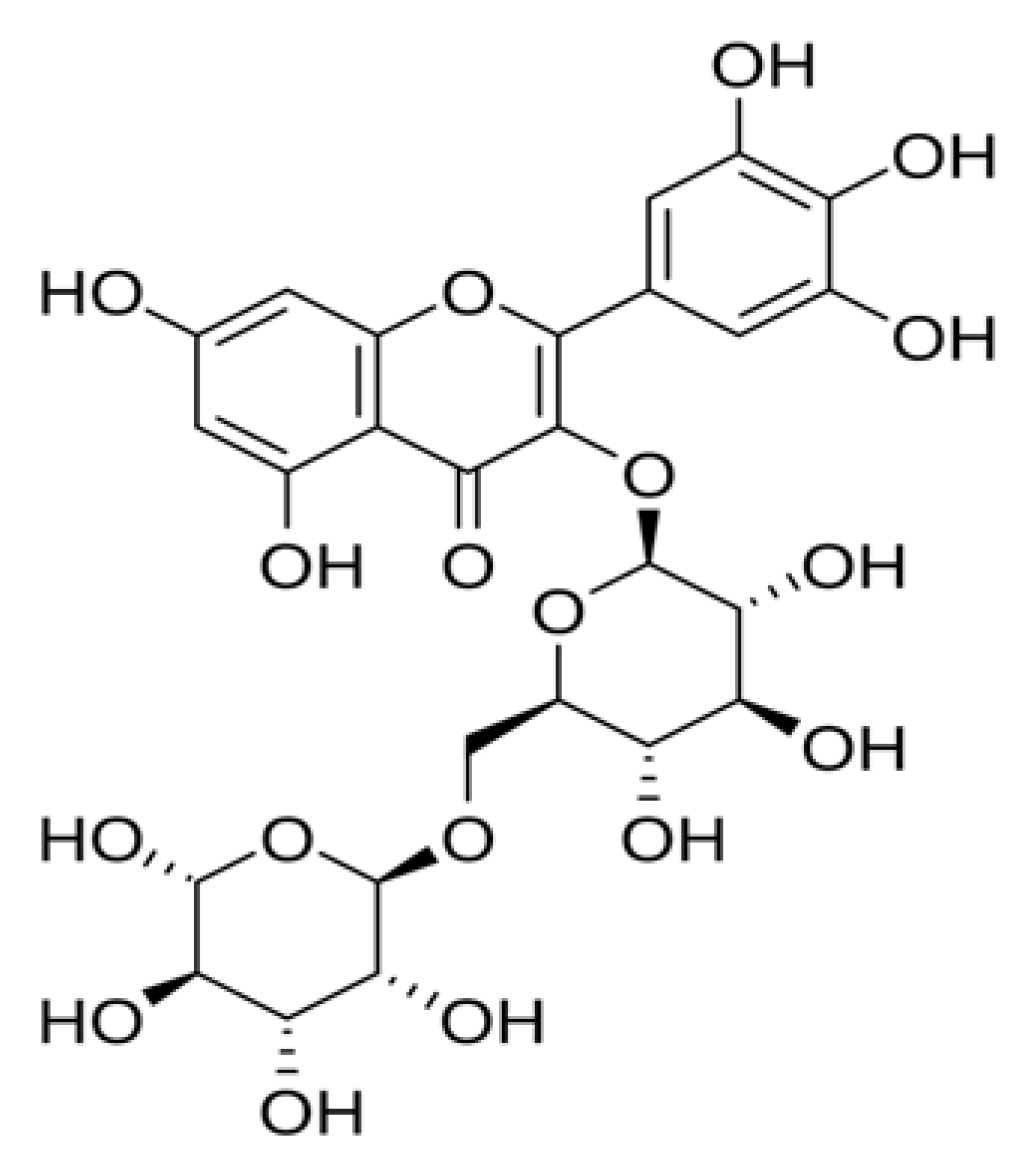
2	3.335	609.33	Quercetin 3-O-rutinoside (Rutin)	[17,18]	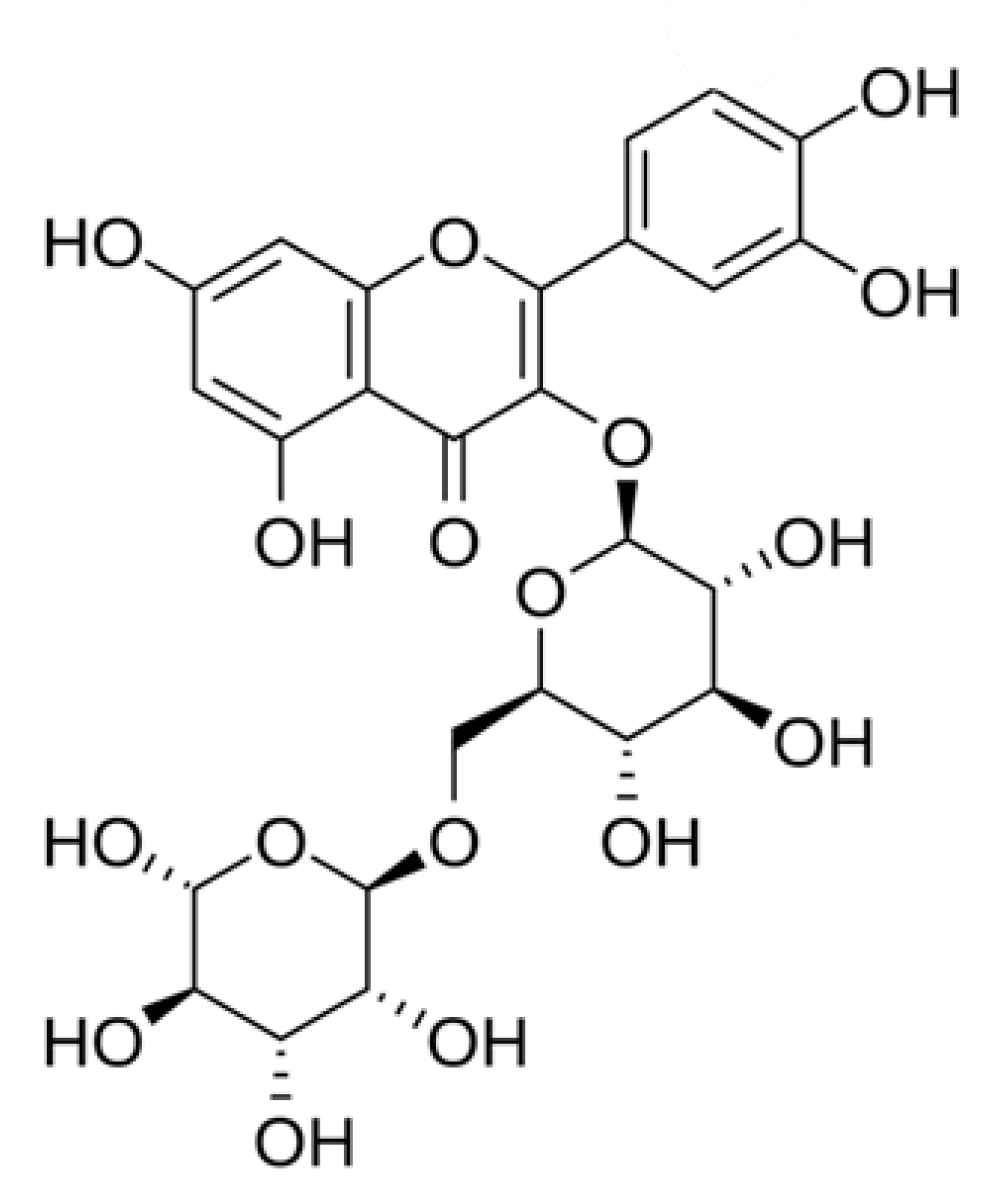
3	3.486	597.40	3′,5′-di-C-β-glucopyranosyl phloretin	[15]	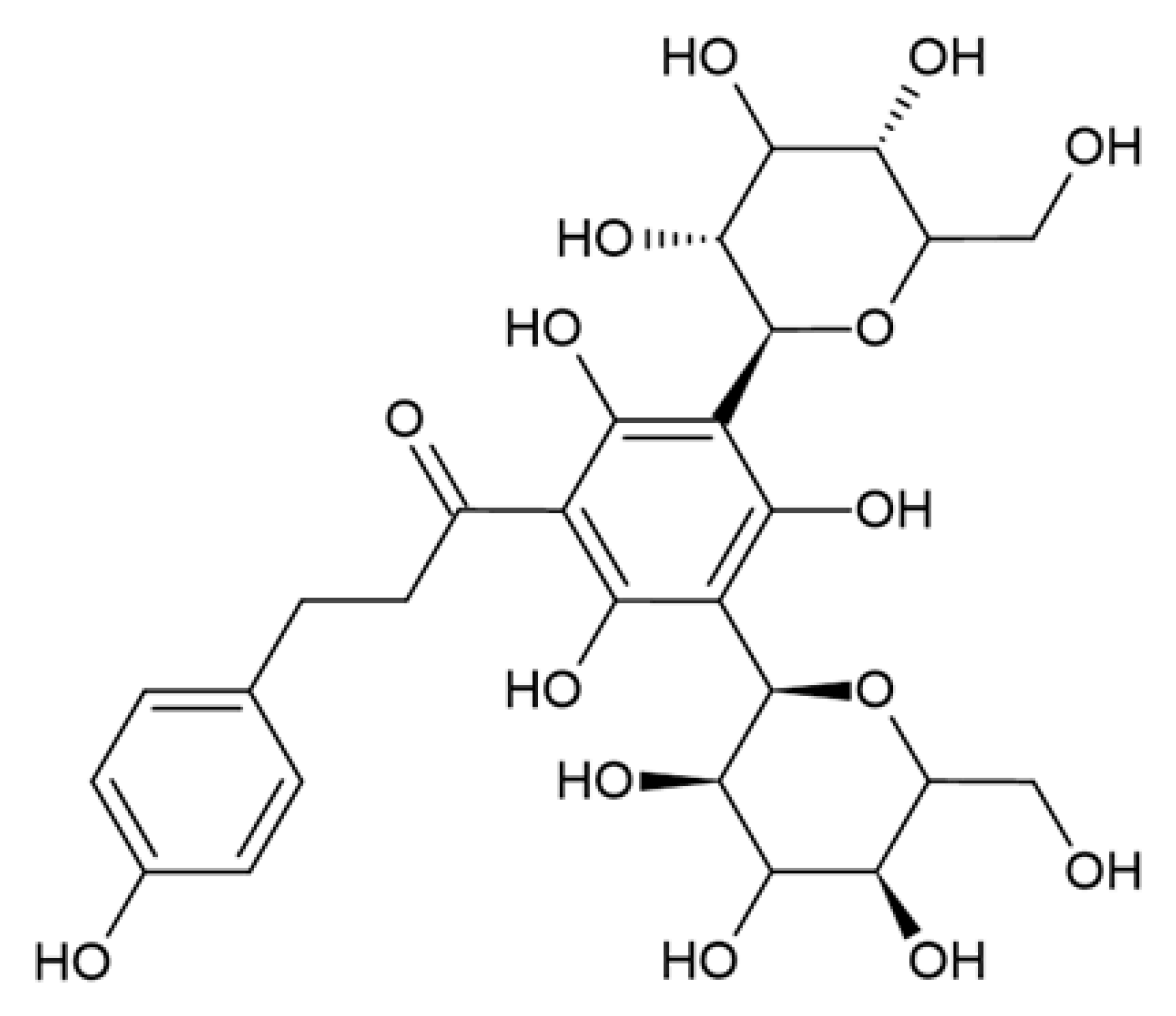
4	3.587	593.38	Kaempferol 3-O-rutinoside (Nicotiflorin)	[16]	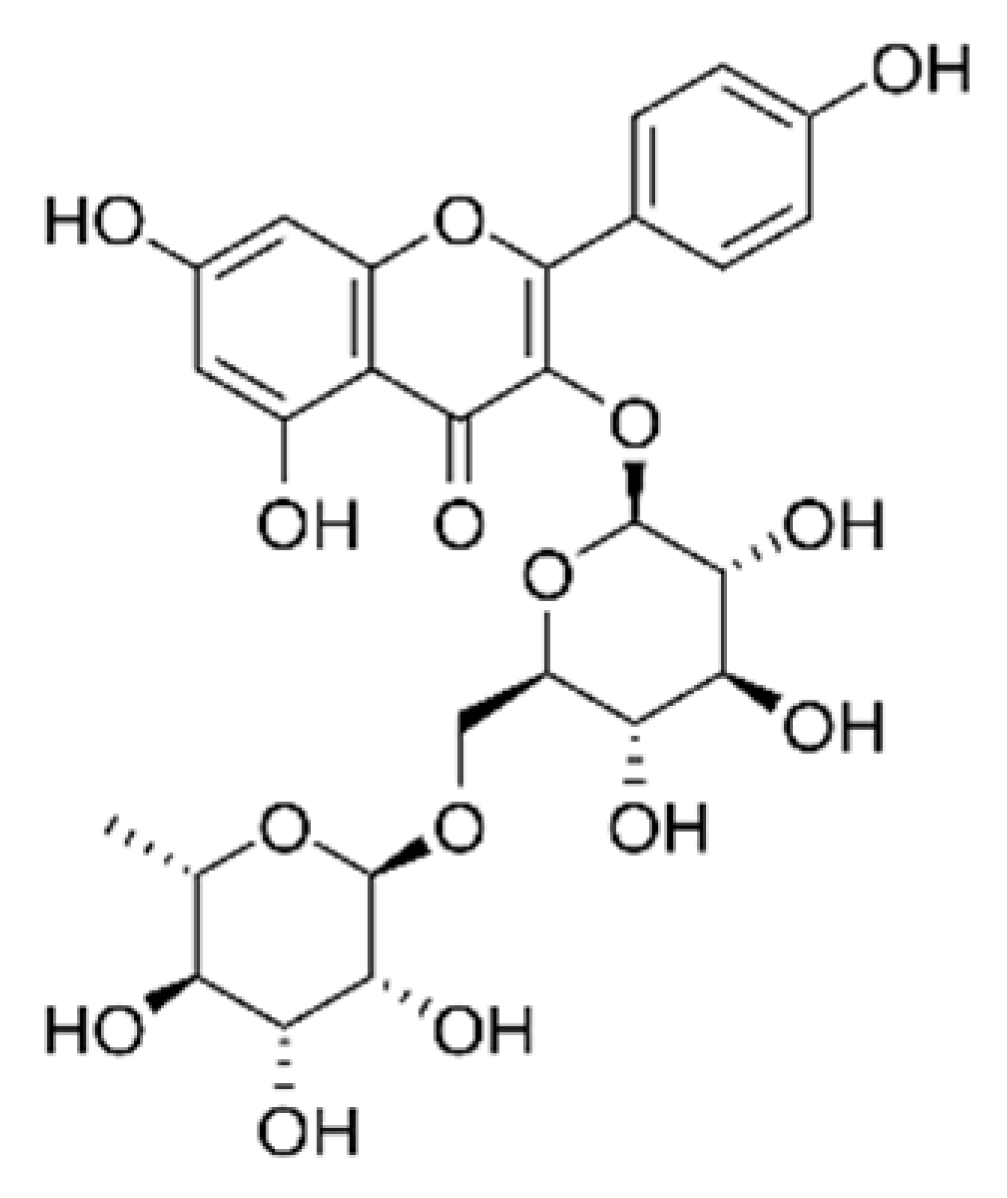

## Data Availability

The data can be made available on request from the corresponding author.

## Abbreviations

The following abbreviations are used in this manuscript:

4-AP	4-aminopyridine
BaCl_2_	Barium chloride
BCB	β-carotene bleaching
[Ca^2+^]i	Intracellular free calcium concentration
CaM	Calmodulin
CCH	Carbachol
cGMP	Cyclic guanosine monophosphate
COX	Cyclooxygenase
DPPH	2,2-diphenyl-1-picrylhydrazyl
EDHF	Endothelium-derived hyperpolarizing factor
Emax	Maximal effect
eNOS	Endothelial nitric oxide synthase
GC	Guanylate cyclase
IC_50_	Half-maximal inhibitory concentration
LD_50_	Median lethal dose
L-NAME	Nω-Nitro-L-arginine methyl ester
MM-PB(GB)SA	Molecular mechanics Poisson-Boltzmann generalized Born surface area
NO	Nitric oxide
ODQ	1H-[1,2,4]oxadiazolo[4,3-a]quinoxalin-1-one
PGI_2_	Prostacyclin
PHE	Phenylephrine
SERCA	Sarco-endoplasmic reticulum Ca^2+^-ATPase
TEA	Tetraethylammonium chloride
UHPLC-ESI-MS	Ultra-high-performance liquid chromatography-electrospray ionization mass spectrometry
VOC	Voltage-operated calcium channels
ZLAqExt	*Ziziphus lotus* (L.) Lam. aqueous extract
